# Gut microbiome alterations in patients with stage 4 hepatitis C

**DOI:** 10.1186/s13099-016-0124-2

**Published:** 2016-09-13

**Authors:** AbdelRahman Mahmoud Aly, AbdelReheem Adel, Ahmed Osama El-Gendy, Tamer M. Essam, Ramy K. Aziz

**Affiliations:** 1 Faculty of Post Graduate Studies for Advanced Sciences, Beni-Suef University, Beni-Suef, 62511 Egypt; 2 Department of Microbiology and Immunology, Faculty of Pharmacy, Beni-Suef University, Beni-Suef, 62511 Egypt; 3 Department of Microbiology and Immunology, Faculty of Pharmacy, Cairo University, Cairo, 11562 Egypt

**Keywords:** Microbiome, Infectious diseases, Virology, Liver disease, Gastro-intestinal tract, High-throughput sequencing, Next-generation sequencing

## Abstract

**Background:**

Hepatitis C virus (HCV) causes debilitating liver diseases, which may progress to cirrhosis and cancer, and claims 500,000 annual lives worldwide. While HCV epidemiology, pathophysiology, and therapy are being deeply studied, rare attention is given to reciprocal interactions between HCV infection , HCV-induced chronic liver diseases, and the human gut microbiome. As Egypt has the world’s highest prevalence of HCV infections, we launched this study to monitor differences in the gut microbial community composition of Egyptian HCV patients that may affect, or result from, the patients’ liver state.

**Results:**

To this end, we analyzed stool samples from six stage 4-HCV patients and eight healthy individuals by high-throughput 16S rRNA gene sequencing using Illumina MiSeq. Overall, the alpha-diversity of the healthy persons’ gut microbiomes was higher than those of the HCV patients. Whereas members of phylum Bacteroidetes were more abundant in HCV patients, healthy individuals had higher abundance of Firmicutes, Proteobacteria, and Actinobacteria. Genus-level analysis showed differential abundance of *Prevotella* and *Faecalibacterium* (higher in HCV patients) vs. *Ruminococcus* and *Clostridium* (healthy group), indicating that the higher abundance of Bacteroidetes in HCV patients is most likely due to *Prevotella* overabundance. The probiotic genus, *Bifidobacterium*, was only observed in the microbiotas of healthy individuals.

**Conclusions:**

To the best of our knowledge, this study provides a first overview of major phyla and genera differentiating stage 4-HCV patients from healthy individuals and suggests possible microbiome remodeling in chronic hepatitis C, possibly shaped by bacterial translocation as well as the liver’s impaired role in digestion and protein synthesis. Future studies will investigate the microbiome composition and functional capabilities in more patients while tracing some potential biomarker taxa (e.g., *Prevotella, Faecalibacterium* vs. *Bifidobacterium*).

**Electronic supplementary material:**

The online version of this article (doi:10.1186/s13099-016-0124-2) contains supplementary material, which is available to authorized users.

## Background

About 130–150 million people suffer from chronic hepatitis C virus (HCV) infection worldwide, and about half a million people die every year because of this resilient virus [1]. Egypt has the highest prevalence of HCV in the world, estimated as 13.9–14.7 % [2, 3]. This means that ~13 million Egyptians are currently infected by HCV.

This virus infects liver cells leading to dramatic physiological and pathophysiological changes. The human liver plays a crucial role in the body’s homeostasis, as most blood proteins are produced by the liver cells (e.g., serum albumin, α-fetoprotein, soluble plasma fibronectin, coagulation cascade and inhibitors of coagulation proteins, and C-reactive protein) [4]. Thus, after HCV infection, the virus starts to destroy hepatic cells, negatively impacting the body’s homeostasis and leading to other dangerous consequences, e.g., chronic liver inflammation, cirrhosis and hepatocellular carcinoma (HCC). HCV patients may experience acute or chronic pathological events after infection. About 15–45 % of HCV patients may clear the virus within 6 months of infection without any treatment; however, 55–85 % will develop chronic HCV infection through 20 years, leading to cirrhosis and HCC [5].

A major part of ongoing research focuses on the causality relationship between HCV infection and its subsequent pathophysiological consequences. Surprisingly though, little is known about the interplay between HCV infection, its complications, and the human gut microbiome [6, 7].

The human body carries 10^14^ microbial cells, outnumbering human cells and contributing about 1.5 kg weight [8]. The genetic content of these microbial communities is estimated at 100× more than human genes, which undoubtedly suggests an important role that the microbiota plays in human health and diseases. For example, gut microbes play a major role in vitamin synthesis, food digestion, and immune system development [9]. Alterations of gut community composition have been linked to several diseases, such as rheumatoid arthritis [10], colorectal cancer [11], obesity [12], depression, anxiety, autism, and others [13]. Furthermore, accruing data suggest a pivotal role for the gut microbiome in the metabolism of drugs, such as acetaminophen, chloramphenicol, digoxin, and sulfasalazine [14].

While a number of studies investigated the role of the gut microbiota in non-alcoholic fatty liver disease [15], alcoholic liver disease [16], liver cirrhosis [17] and hepatocellular carcinoma [18], much less attention was given to the mutual interactions between viral hepatitis, its complications, and the human microbiome. A handful studies considered HCV complications and their relation to the integrity of gut bacteria as well the immune system [e.g. 19, 20]; however, to the best of our knowledge, no study has investigated the gut microbiome composition of chronic HCV patients, and whether it differs from healthy individuals [6]. Accordingly, we launched this study to explore the composition of the gut microbiomes of HCV patients at Beni-Suef Emergency Hospital, (Beni-Suef, Egypt) in comparison to healthy individuals from the same city.

## Methods

### Ethical considerations

Informed consent was obtained from all subjects before collection of stool samples. All procedures were approved by the ethics committee of Faculty of Pharmacy, Cairo University.

### Study design and subjects

This study is a prospective case–control study. Samples were taken from seven HCV patients and eight healthy individuals. All patients and healthy volunteers were adult males, aging between 21 and 65. Exclusion criteria included: inflammatory bowel syndrome, colorectal cancers or any systemic antibiotic use during or 3 months prior to the study period initiation. Essential liver functions about the patients were collected. Other necessary information about other heart, chest, or kidney diseases, hypertension, and other major types of hepatitis were provided (Table 1; Additional file 1: Table S1), and any patients who failed these criteria were excluded.

**Table 1:** Information about patients and healthy volunteers from whom stool samples were collected

ID	Status	HCV infection	HCV- qPCR count	ALT	AST	Serum albumin	Bilirubin total	Bilirubin direct	Creatinine	Prothrombin time (PT)	Hemoglobin %	Hepatitis stage	Liver cirrhosis stage
P1	Patient	Yes	29,442	(N:12)21	(N:12)16	3.8	0.7	N/A	1	13.1	14.5	Stage 4	Stage B
P2	Patient	Yes	93,100	(N:50)79	(N:37)82	2.9	0.91	0.3	0.7	14.7	12.4	Stage 4	Stage B
P3	Patient	Yes	19,753	N/A	N/A	N/A	N/A	N/A	1.4	N/A	N/A	Stage 4	Stage B
P4	Patient	Yes	552,296	(N:50)25	(N:37)36	4.4	0.59	0.18	N/A	17.2	15.6	Stage 4	Stage B
P5	Patient	Yes	2,026,000	(N:50)90	(N:37)102	3.8	0.9	0.2	1.1	11.6	11.7	Stage 4	Stage B
P6	Patient	Yes	645,599	(N:50)38	(N:37)35	4.5	0.62	0.31	1.1	11.5	15.8	Stage 4	Stage B
P7	Patient	Yes	226,566	(N:12)16	(N:12)18	4.4	0.8	N/A	N/A	11.3	14	Stage 4	Stage B
H1	Healthy	No	–	N/A	N/A	N/A	N/A	N/A	N/A	N/A	N/A	–	–
H2	Healthy	No	–	N/A	N/A	N/A	N/A	N/A	N/A	N/A	N/A	–	–
H3	Healthy	No	–	N/A	N/A	N/A	N/A	N/A	N/A	N/A	N/A	–	–
H4	Healthy	No	–	N/A	N/A	N/A	N/A	N/A	N/A	N/A	N/A	–	–
H5	Healthy	No	–	N/A	N/A	N/A	N/A	N/A	N/A	N/A	N/A	–	–
H6	Healthy	No	–	N/A	N/A	N/A	N/A	N/A	N/A	N/A	N/A	–	–
H7	Healthy	No	–	N/A	N/A	N/A	N/A	N/A	N/A	N/A	N/A	–	–
H8	Healthy	No	–	N/A	N/A	N/A	N/A	N/A	N/A	N/A	N/A	–	–

All subjects were non-smokers. They had no reported hypertension, heart, kidney, or chest diseases. None of the sampled subject had reported hepatitis A, B or HIV (details in Additional file 1: Table S1)

*ALT* alanine transaminase enzyme; *AST* aspartate transaminase enzyme; *N/A* data not available

### Sample collection and DNA extraction

Stool samples were collected in sterilized containers and immediately stored at −20 ℃. On the day after the stool samples were collected, DNA was extracted by the QIAamp DNA Stool Mini Kit (Qiagen, Valencia, CA), and the extracted DNA samples were aliquoted and stored at −80 ℃.

DNA quality and quantity were measured by NanoDrop 2000 UV–Vis spectrophotometer (Thermo Fisher Scientific, Waltham, MA). Concentrations ranged between 50 and 200 ng/µl. In addition, polymerase chain reaction (PCR) amplification of 16S rRNA gene (using universal primers—27F, 1492R) followed by gel electrophoresis was used to confirm that the samples contained prokaryotic DNA. Out of 15 DNA specimens, only one (from a HCV patient) failed QC; thus, the rest of the study was conducted with DNA from six patients and eight healthy controls.

### 16S rRNA amplification of V4 region and illumina sequencing

Sequencing was performed at the Pacific Northwest National Laboratory (PNNL). The hypervariable region V4 of the 16S rRNA gene was amplified using forward primer (515f) and reverse primer (806r). Subsequently, 12 bp unique barcode sequences were attached to each sample for multiplexing. PCR amplification of the V4 region of the 16S rRNA gene was performed according to the Earth Microbiome Project protocol (http://www.press.igsb.anl.gov/earthmicrobiome/emp-standard-protocols/16s/), as described by Caporaso and coworkers [21], except that the 12-bp barcode sequence was included in the forward primer. Amplicons were sequenced in an Illumina MiSeq using the 300 cycle MiSeq Reagent Kit v2 (http://www.illumina.com/) according to manufacturer’s instructions.

### Bioinformatics analysis

The QIIME software suite (v 1.9.1) was used for sequence analysis and primary statistics [22]. Different QIIME scripts were used as follows. First, the validate_mapping_file.py script was used for mapping file validation, join_paired_ends.py for merging paired-end read data, split_libraries.py for quality filtering and demultiplexing the samples.

Afterwards, we followed the de novo as well as the closed-ref operational taxonomic unit (OTU) picking protocols to assign OTUs to our reads. The Greengenes database (V 13.8) [23] was used as a reference for the closed protocol. Taxonomic composition (phylum, class, family, etc.) was assigned by the summarize_taxa_through_plots.py script.

We calculated the alpha diversity using alpha_diversity.py script (Chao1, Shannon, Simpson, Phylogenetic Diversity, PD, and Observed OTUs metrics) and the beta diversity by beta_diversity.py script. Final data analysis and graphs were generated by the PhyloSeq package of the R project [24] as well as ggplot2 and reshape2 packages. LEfSe [25] was used for biomarker discovery analysis.

## Results

The fecal microbiomes of six HCV patients and eight healthy individuals (Table 1; Additional file 1: Table S1), from the city of Beni Suef in Egypt, were sequenced by high-throughput Illumina MiSeq. High-throughput sequencing generated 651,912 reads that passed initial quality filtering (median reads per sample = 46,219; median read length = 253 bp). The quality of these sequence data was checked by standard quality control procedures, (e.g., total number of input sequences; barcode not in mapping file; reads too short after quality truncation; count of N characters exceeding limit; Illumina quality digit; barcode errors exceeding max; median sequence length), and the filtered data that passed quality control were used for analysis (primary taxon assignments are provided in Additional file 2: Table S2).

Even though some samples had much fewer reads than others, rarefaction curves indicated that the sampling depth was sufficient for data comparison, as all samples reached an asymptote (Additional file 3: Figure S1).

### Primary data validation

At first, we sought to validate the raw 16S sequence data sets. In the absence of robust published high-throughput data specifically representing Egyptian gut microbiome composition, we chose to compare our samples to well-documented and published gut microbiome sequences, such as the American Gut samples (available from https://www.github.com/biocore/American-Gut), to validate the results of taxonomic assignments in our samples. Although those samples are from individuals living in different geographical area and consuming different type of diet, the data set is still a valid representative of human gut microbes.

The comparison of our 14 samples to the American Gut samples showed that the major taxa usually associated with the human colon environment (e.g., phyla Firmicutes and Bacteroidetes; genera *Faecalibacterium*, *Ruminococcus*, and *Bacteroides*) were shared by both data sets—yet in different proportions (Additional file 3: Figures S2, S3). For example, the overall Firmicutes-to-Bacteroides ratio in the Egyptian samples was 1.1 (vs. 1.4 in the American Gut samples).

### Alpha-diversity analysis

**Fig. 1:**
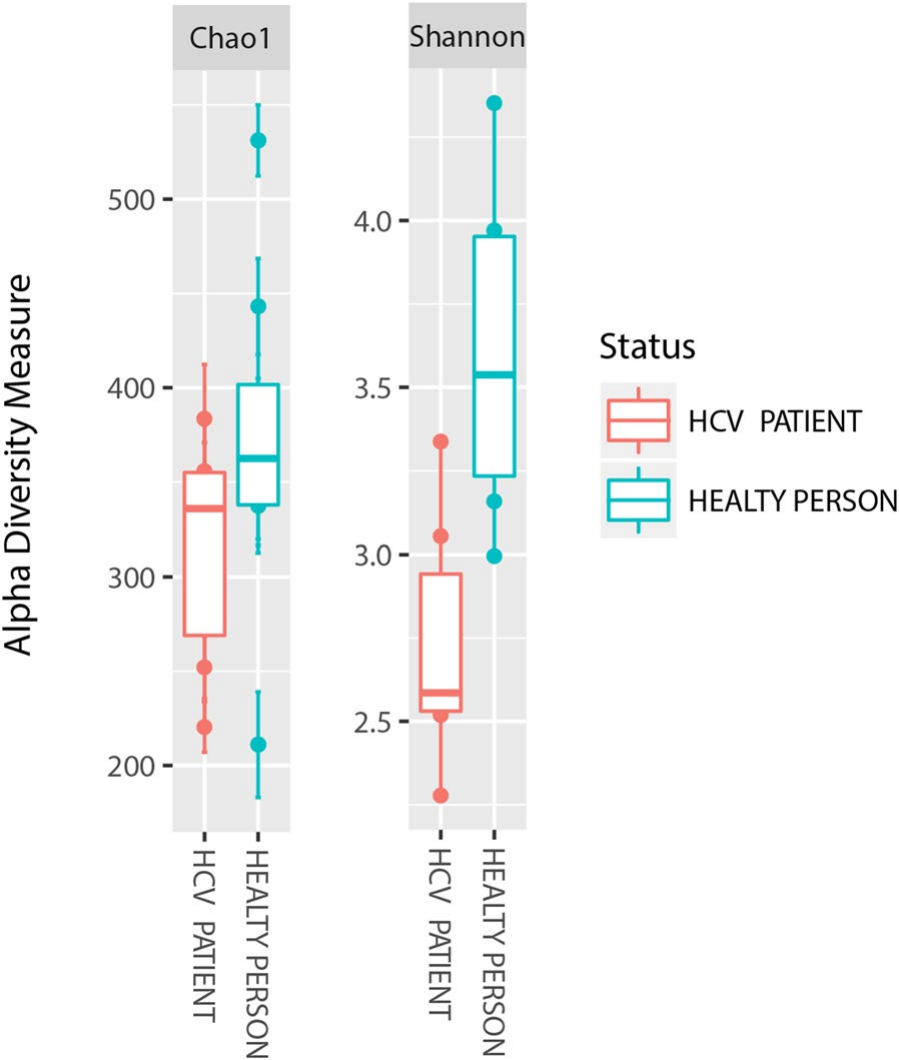
Alpha diversity estimation in patients vs. control group (Chao and Shannon indexes)

Using different diversity indices (e.g., Chao, Shannon, or Simpson), we found that, regardless of the used metric, the richness and diversity of healthy individuals’ microbiota were higher than those of HCV patients (Fig. 1; Additional file 3: Figure S4). This finding agrees with several other human microbiome studies in which chronic inflammation tends to decrease biodiversity at different microbiome sites [26–28].

### Distinct core microbiomes differentiate healthy controls from HCV patients

**Fig. 2:**
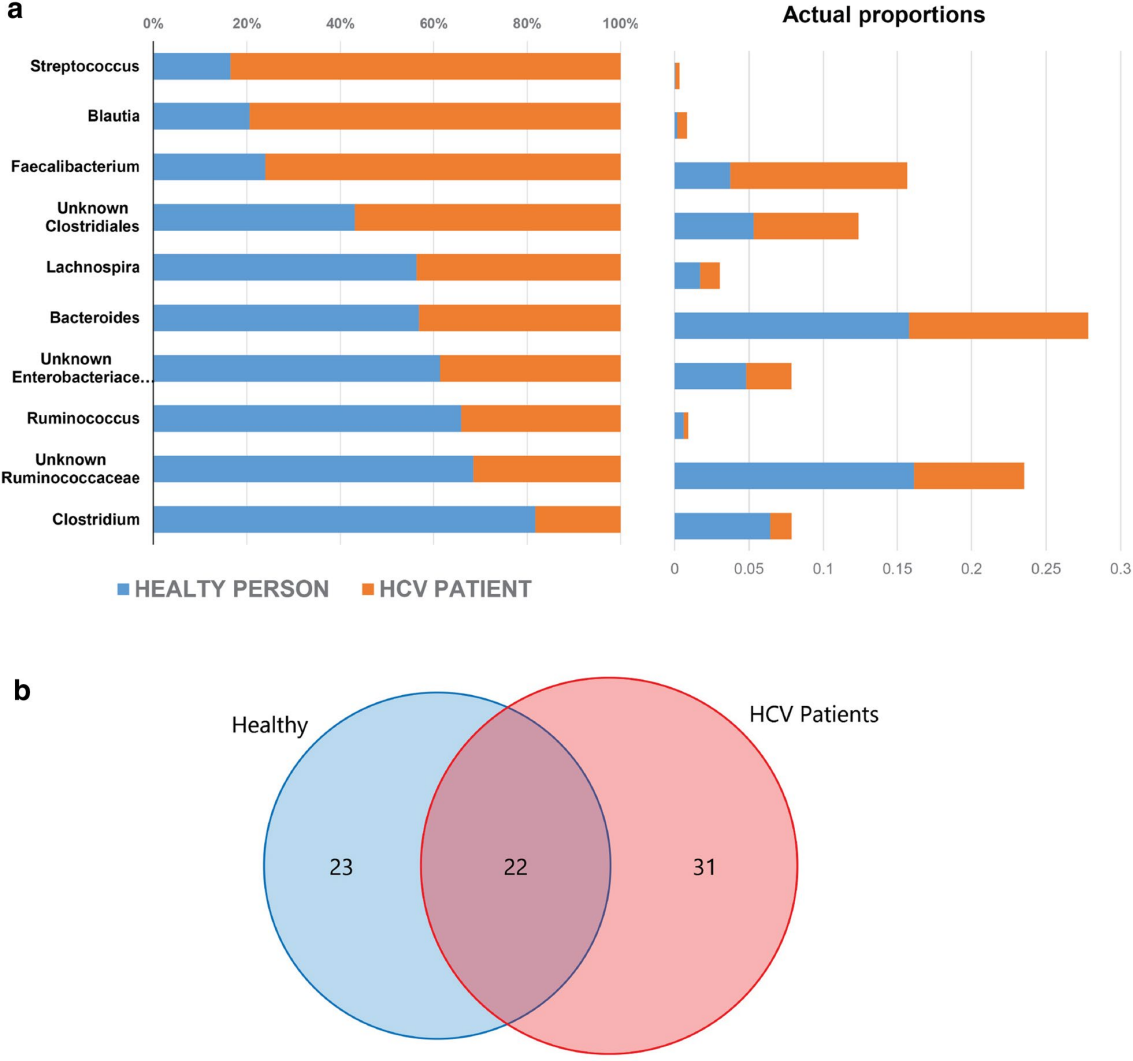
Core microbiomes of analyzed samples. **a** Main taxa of the core microbiome of all fecal samples and their relative distribution in healthy controls (*blue*) compared to patients with HCV (*orange*). *Left panel* average relative abundance of 16S sequence reads representing core taxa (in percent). *Right panel* actual values of the average proportions of 16S sequence reads representing core taxa per group. **b** Venn diagram representing the core OTUs (genus level) in each analyzed group and their intersection

Core bacterial taxa shared by each group (healthy controls and HCV patients) were identified. Overall, 22 distinct OTUs were conserved among all samples, constituting a core gut microbiome. This core set is characterized by genera *Streptococcus*, *Ruminococcus, Clostridium, Faecalibacterium, Bacteroides*, *Blautia*, and *Lachnospira* in addition to some undefined members of families Enterobacteriaceae and Clostridiaceae. Most of these genera were differentially distributed among healthy and HCV patients (Fig. 2a). Overall, 22 OTUs were shared by both groups, 23 distinguished healthy controls, and 31 distinguished HCV patients (Fig. 2b).

### Major taxonomic differences between microbiomes of healthy individuals and HCV patients

The ultimate goal of this study was identifying consistent differences in microbial composition between the two analyzed groups (healthy controls and HCV patients).

**Fig. 3:**
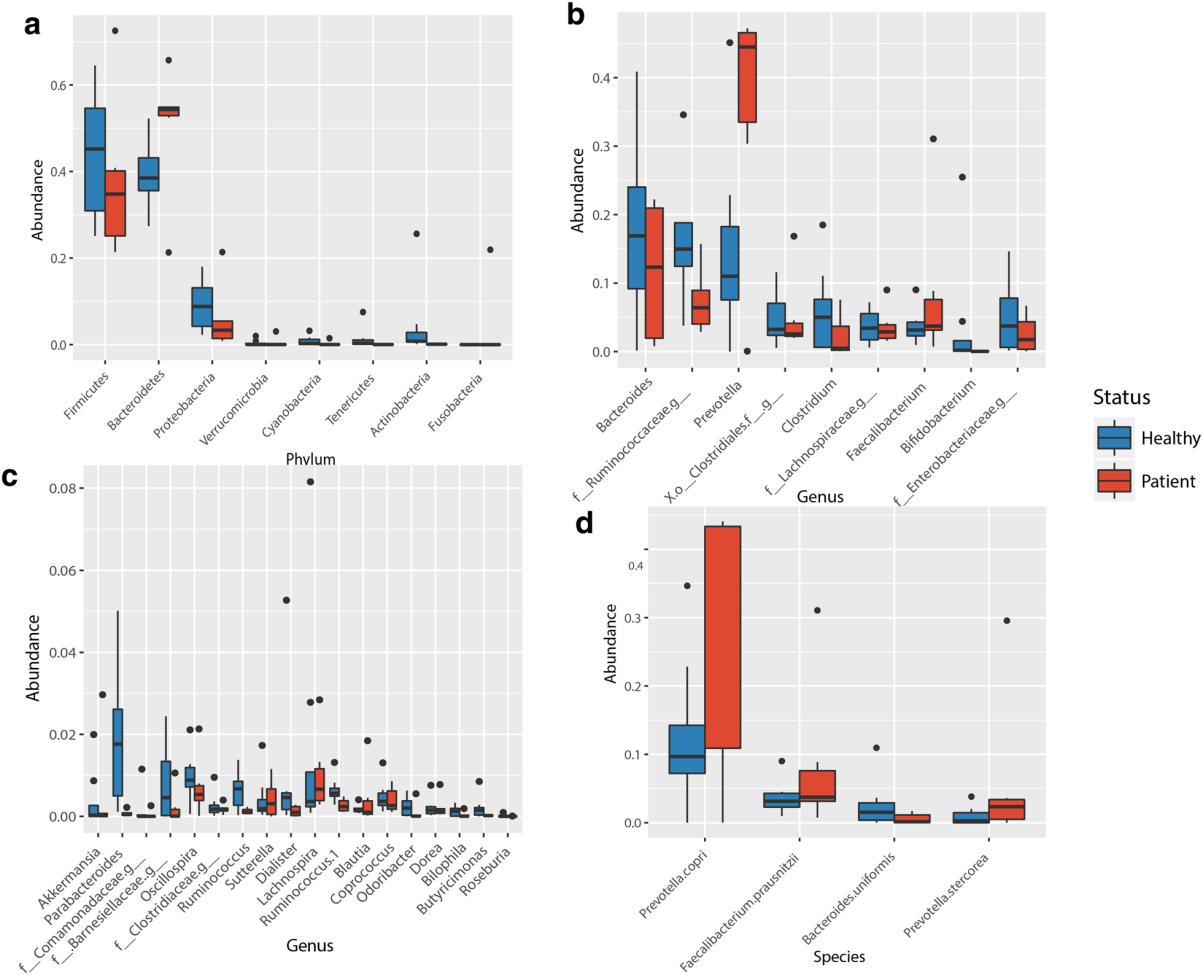
*Boxplots* representing the average proportion of each 16S sequence read attributed to each taxon between the two groups (*Blue* healthy control samples; *Red* patient samples). **a** On the phylum level, **b** on the genus level—major taxa; **c** on the genus level—minor taxa; **d** on the species level—selected taxa

On the phylum level, a mild but significant increase was observed in the ratios of Bacteroidetes among HCV patients (Kruskal–Wallis *p* = 0.039), whereas Firmicutes were slightly more abundant in healthy controls (Fig. 3a; Additional file 3: Figure S5); yet that observed overabundance of Firmicutes is not statistically significant (Kruskal–Wallis *p* = 0.301).

Genus-level analysis, however, was more informative (Fig. 3b, c; Table 2). It revealed that genus *Prevotella* was clearly enriched in HCV patients (*p* = 0.038), possibly inflating the total Bacteroidetes abundance observed on the phylum level. Other minor genera that were also significantly overabundant in HCV patients are *Acinetobacter*, *Veillonella*, and *Phascolarctobacterium* (Table 2). In addition, *Faecalibacterium* was another genus with higher abundance in HCV patients than in healthy controls; yet, *Faecalibacterium* abundance was less consistent among HCV patients. On the other hand, genera *Ruminococcus* and *Clostridium* were more abundant in healthy controls (Fig. 3b, c; Additional file 3: Figure S6). Interestingly, two of the healthy controls had relatively high abundance of the probiotic genus, *Bifidobacterium*, which was undetected in any of the HCV patients.

**Table 2:** Genera that are statistically significantly different between the two groups (non-parametric *t* test)

Genus	Control mean	HCV patients mean	Ratio	*p* value
Higher in HCV patients				
*Acinetobacter*	0	0.0001	N/A	0.003
*Prevotella*	0.1501	0.3555	0.42	0.038
*Veillonella*	0.0006	0.0107	0.06	0.056
*Phascolarctobacterium*	0.0012	0.0158	0.08	0.057
Higher in healthy controls				
*Ruminococcus*	0.0064	0.0027	2.37	0.006
*Parabacteroides*	0.0190	0.0007	27.14	0.021
*Butyricimonas*	0.0021	0.0002	10.5	0.056

Some other sample-specific peculiarities are worth mentioning. For example, one patient had a fair amount of phylum Fusobacteria, which has been described as a biomarker of colon cancer [29]. Another individual exhibited an unusual overabundance of phylum Actinobacteria (Additional file 3: Figure S5).

**Fig. 4:**
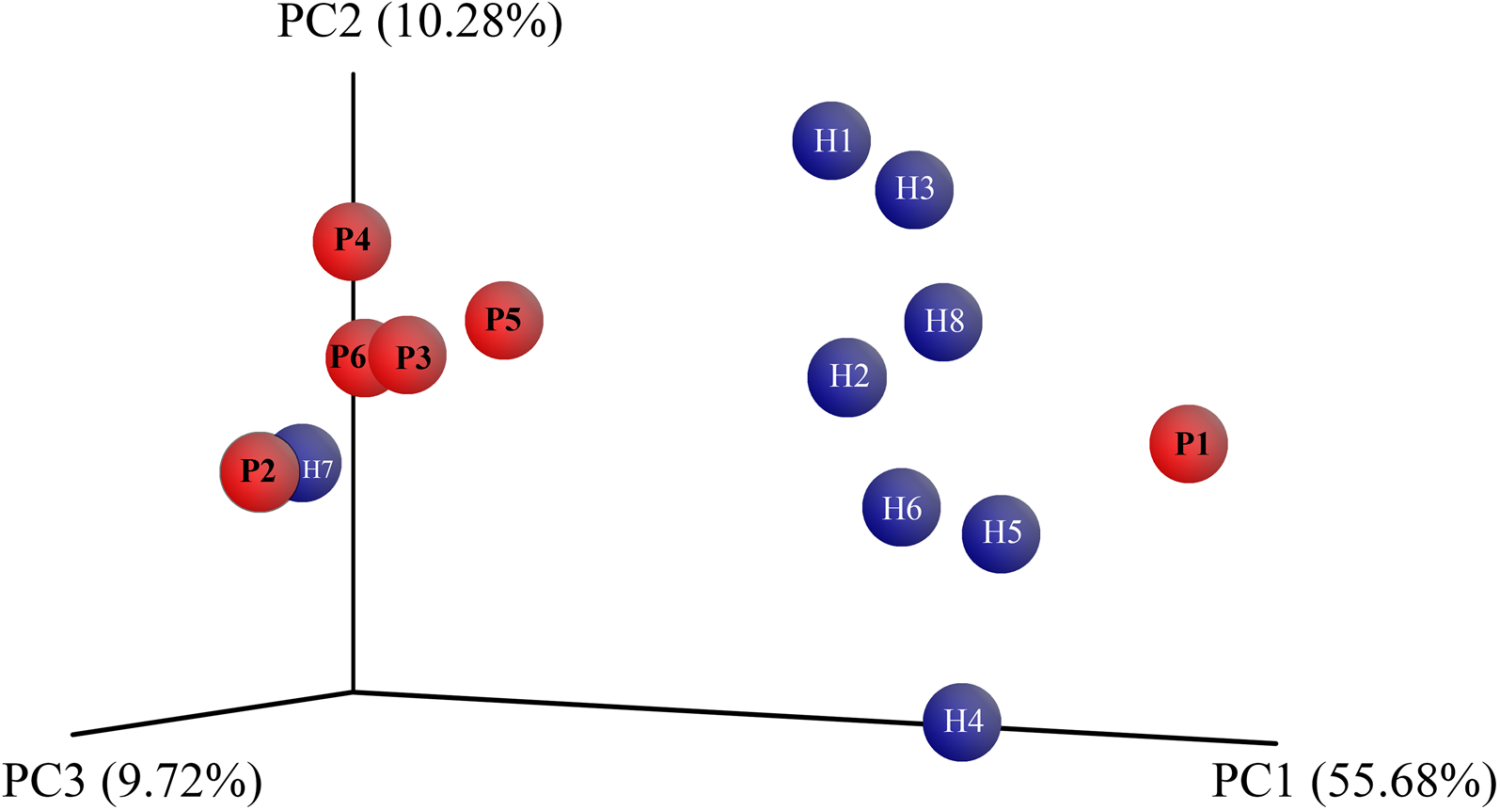
Principal coordinate analysis representing the beta diversity estimated by the weighted UNIFRAC method [30]. Each* sphere* represents one sample (*Blue* healthy control, H1–H8; *Red* patients with HCV, P1–P8). The three principal coordinates (PC1 through PC3) explain 55.68, 10.28 and 9.72 %, respectively

In sum, although only one single phylum was statistically significantly different between patients and controls, and although only three genera were clearly differential, the OTU differences were sufficient to separate most cases into two distinct clusters, as revealed by principal coordinate analysis (Fig. 4). All patients, except P1, clustered together, while all healthy controls but H7 clustered together.

The clustering was mostly affected by the relative abundance of *Prevotella*, since patient 1 (P1) coincidentally had no detectable *Prevotella* OTUs while healthy control 7 (H7) had an unusually higher proportion than the rest of the healthy control group.

However, this patient (P1)—in particular—had the highest proportion of *Faecalibacterium*, possibly suggesting that the combined proportion of *Prevotella* and *Faecalibacterium* may be a good biomarker/predictor of the HCV-associated microbiome.

**Fig. 5:**
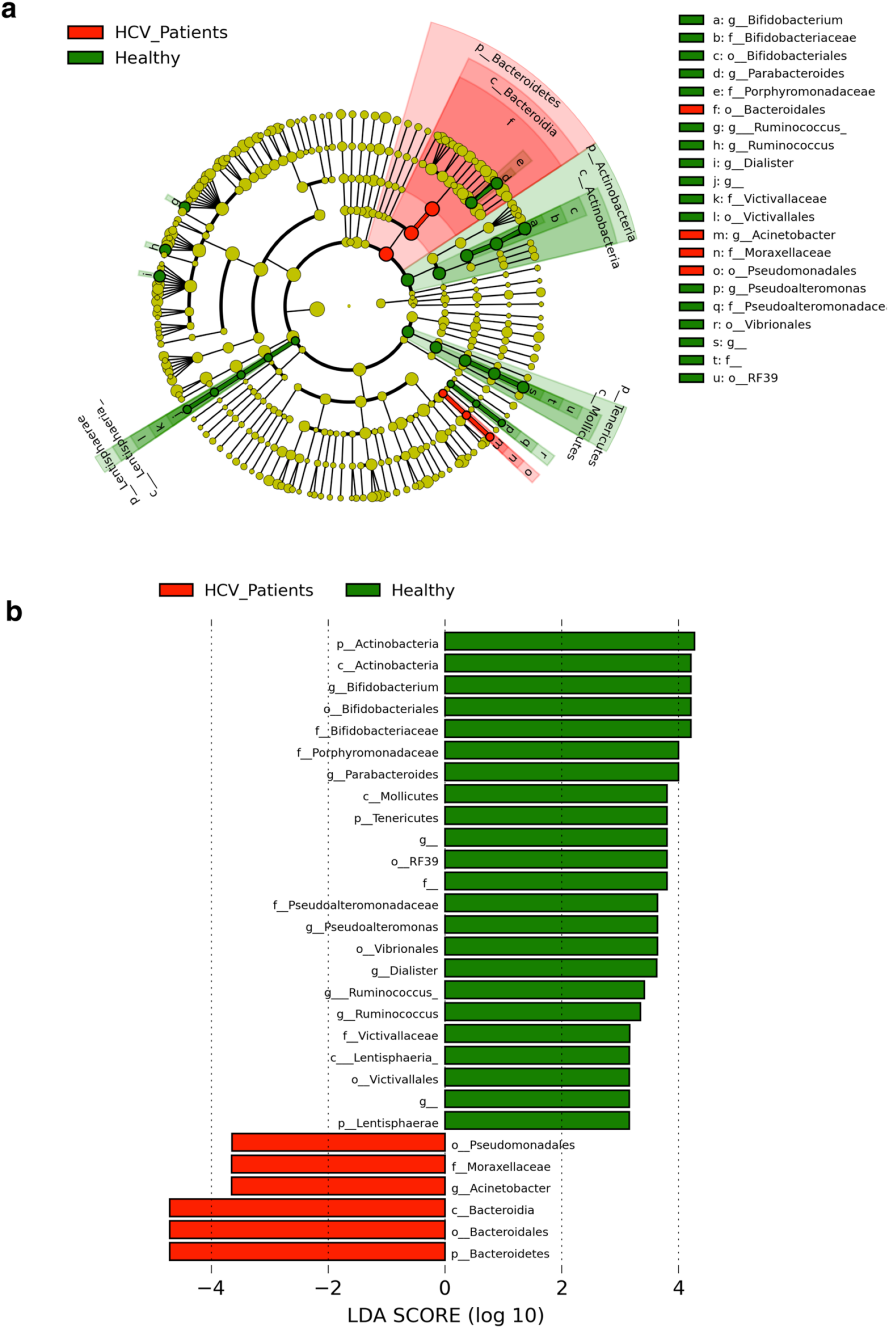
LEfSe classification analysis

To run a full, unbiased investigation of which OTUs can serve as biomarkers, we used the LEfSe classification tool. This analysis was able to pick some of the minor OTUs, which were not as obvious in the taxon chart analyses (Fig. 3; Additional file 3: Figures S5, S6), and defined a list of taxa as potential biomarkers for the healthy vs. HCV groups. For example, the bacteroidetes can serve as biomarkers for HCV patients on the phylum, order, and class level while a few taxa were markers of the healthy microbiome, most prominent of which are genera *Bifidobacterium*, *Ruminococcus* and phylum Tenericutes (Fig. 5).

## Discussion

Over the past few years, the advancement of high-throughput sequencing technologies led to astonishing discoveries about the microbial communities that live in and on the human body, the human microbiota. The human gut contains trillions of microbial cells, many of which are metabolically active. As 70 % of the liver blood access is derived from the gastrointestinal tract (GIT) through the portal vein, it is expected that compositional changes in the gut microbial community will affect the liver physiological state (gut-liver axis). On the other hand, the liver has major impact on digestion and, thus, the liver health and status will directly impact the intestinal environment and its resident microbes.

Surprisingly, little is known about the gut microbiome of HCV patients [6, 7], although the disease has >2 % global prevalence. Given the chronic nature of the disease, and its unusually high prevalence in Egypt, we sought to explore the gut microbiome of HCV patients with no other underlying disease, in comparison with healthy controls from the same geographical area (having similar diet and lifestyle). To this end, we sequenced and analyzed the microbial community structure in six HCV patients and eight healthy controls, and we observed a few consistent differences. Genera *Prevotella* and *Faecalibacterium* were more enriched in HCV patients in addition to the minor genera *Acinetobacter*, *Veillonella*, and *Phascolarctobacterium*, while *Ruminococcus*, *Bifidobacterium*, and some clostridia were more abundant among healthy controls.

In agreement with several other microbiome studies, a higher microbial diversity was observed in fecal communities of healthy controls than in the patient group. These patients were not in-patients and thus were not in a protected environment or under limited dietary options to suggest that this decrease in diversity is a consequence of hospitalization. More likely, the lower diversity is a result of complex factors: one major factor, disruption of homeostasis caused by the chronic HCV infection, may have induced a state of dysbiosis in the intestine. Additionally, the immune system’s adaptation to the state of chronic infection may be another major factor in decreasing gut microbial diversity. Cytokines, IgA levels, and T cell mobilization are all possible factors that control that diversity.

**Fig. 6:**
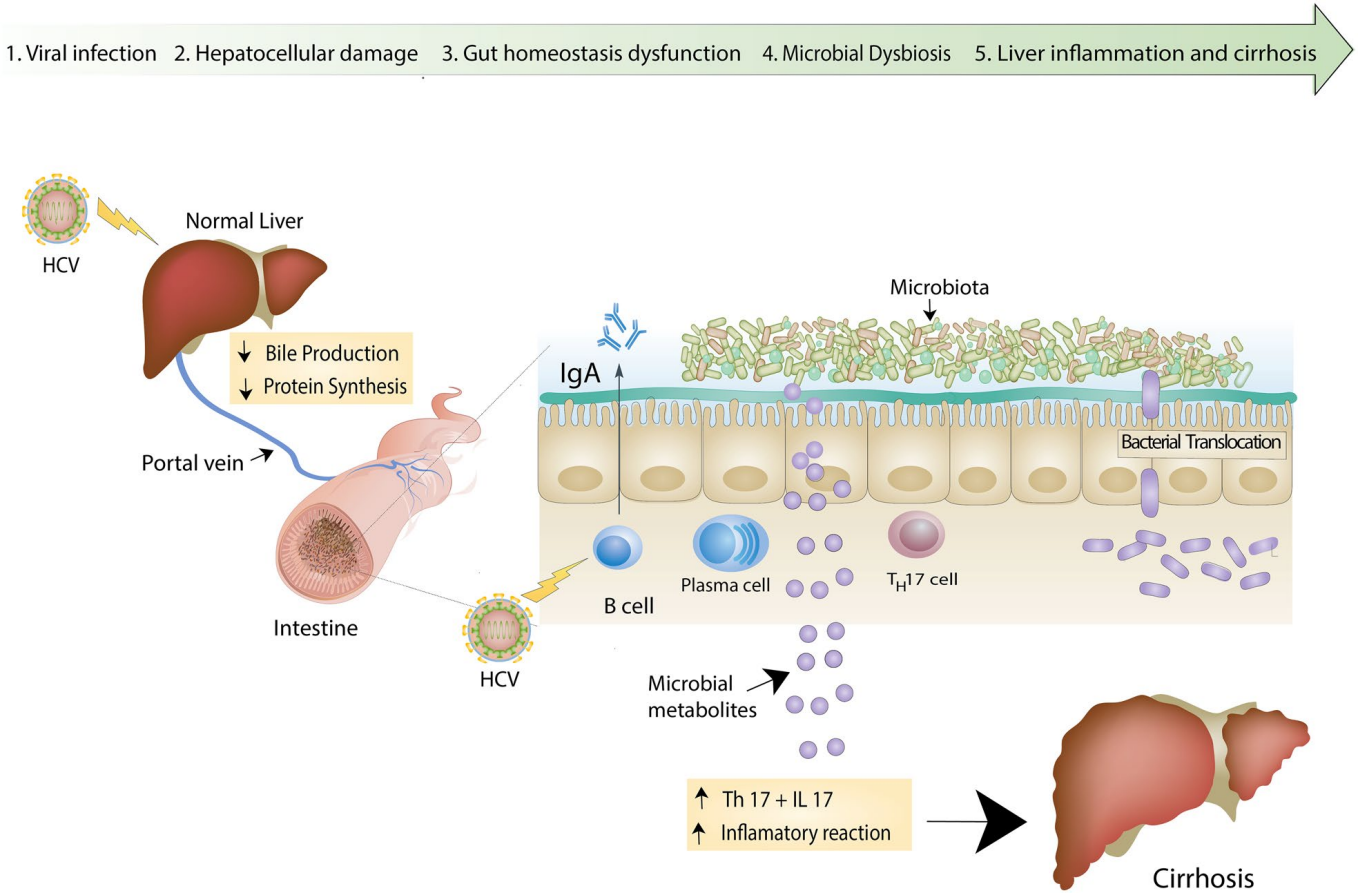
Suggested gut-liver axis model for chronic HCV infection. HCV starts to invade hepatocytes with massive destruction of the hepatocyte architecture and structure (1), which leads to their loss of functions, such as bile salt production and protein synthesis (2). Consequently, such damage leads to disruption of the gut homeostasis and environmental changes (3), which can affect and change the microbiota abundance and composition (possible dysbiosis, 4). The overabundance of microbes, such as *Prevotella*, may produce signaling microbial metabolites that may influence and initiate the inflammatory mediators leading to liver inflammation and cirrhosis (5). This model is based on an integration of the findings of this study with literature [19, 20, 35–38, 42, 43, 45]

An interesting characteristic of HCV is that the virus invades gastric cells as both of the liver and gastric cells share common embryogenic origin. Moreover, HCV infects the gastric B-lymphocytes, which produce IgA antibodies [31]. IgA is known to modulate the gut microbiome composition and abundance [32], possibly behind the higher than average ratios of *Prevotella* and *Paraprevotella* in the HCV patients’ samples (Fig. 6).

Perhaps the most significant influence of HCV on the gut microbiome is related to the pathophysiological alterations of the liver, eventually interfering with its digestive functions. For example, HCV infection leads to low bile production, subsequently leading to bacterial overgrowth and changes in gut microenvironment and microbial community [33–38]. Another interesting finding, previously reported as a link between the gut and the liver in cirrhotic patients, is bacterial translocation. Bacterial translocation is the migration of gut bacteria or their products to mesenteric lymph nodes or possibly to other organs, including the liver [28, 39]. Causes of bacterial translocation include immune dysfunction, alteration of the luminal factors and altered intestinal permeability. A direct link between viral hepatitis and bacterial translocation has not been established, but studies suggested that the degree of liver disease in patients with HCV might be associated with microbial translocation [19], and bacterial translocation was indeed observed in chronic HCV patients [20]. Thus, it is not unlikely that liver damage and subsequent alterations in homeostasis as well as reduction in biliary secretion may be causing bacterial translocation, as we suggest in our hypothesized model (Fig. 6). Future studies should address this link in more depth.

One more possible reason behind the alteration in microbial composition, particularly the overabundance of *Prevotella* in HCV patients, is the dietary carbohydrate intake. In healthy individuals, high carbohydrate intake has been associated with expansion of *Prevotella* [40]. In HCV patients, it is possible that impairment of digestion and absorption may lead to higher carbohydrates concentrations in small and large intestine, and consequently expansion of *Prevotella*.

Last but not least, *Prevotella copri* abundance has been correlated with Th17 and IL-17 (inflammatory mediator), which are reportedly at high levels in HCV patients [41, 42]. In support of our hypothesized model (Fig. 6), a very recent study used a novel probiotic mixture to slow down HCC growth in mice through suppression of Th17 cells and IL-17 [43].

HCV patients in this study have higher abundance of *Faecalibacterium prausnitzii* in their fecal microbiomes (Fig. 3d), which has been described to have anti-inflammatory effects [44]. It is not clear which mechanism drives the expansion of *F. prausnitzii* in some patients but not in others; however, most likely it is related to the relative cytokine levels in the intestinal environment.

## Conclusions

In conclusion, we analyzed the fecal microbiomes of six patients with stage 4 HCV infection in comparison to eight healthy individuals from the same city and validated the data by comparing them to a larger data set randomly selected from the American Gut samples. Patients with HCV had a few significant changes that may be related to liver-controlled homeostasis, protein synthesis, lipid digestion, or possibly to bacterial translocation, immune modulation, or a combination of all of the above mechanisms.

Based on our findings and on literature, we suggest a brief model (Fig. 6) that could explain the changes we observed in microbiota composition. This model can serve as a working hypothesis for future studies with larger number of samples from more individuals, and/or deeper analysis of metagenomic sequence reads. The role of *Prevotella*/*Faecalibacterium* vs. *Ruminococcus*/*Bifidobacterium* relative abundance as biomarkers for chronic HCV infections, or disease progression, is worth further investigations.

## Additional files

10.1186/s13099-016-0124-2 Information collected about patients and healthy volunteers from whom stool samples were collected.
10.1186/s13099-016-0124-2 Number 16S reads assigned to different operational taxonomic units (OTUs).
10.1186/s13099-016-0124-2 Supplementary figures.

## Abbreviations

GIT: gastrointestinal tract
HCC: hepatocellular carcinoma
HCV: hepatitis C virus
OTU: operational taxonomic unit
PCR: polymerase chain reaction
