# Supplementary material for: Gut microbiome alterations in patients with stage 4 hepatitis C
Source: Gut Pathog. 2016 Sep 13;8(1):42. doi: 10.1186/s13099-016-0124-2 (PMC5020480; doi:10.1186/s13099-016-0124-2)
Supplement: Supplementary file 3 — 10.1186/s13099-016-0124-2 Supplementary figures. [file 13099_2016_124_MOESM3_ESM.pdf]

Supplementary Figures

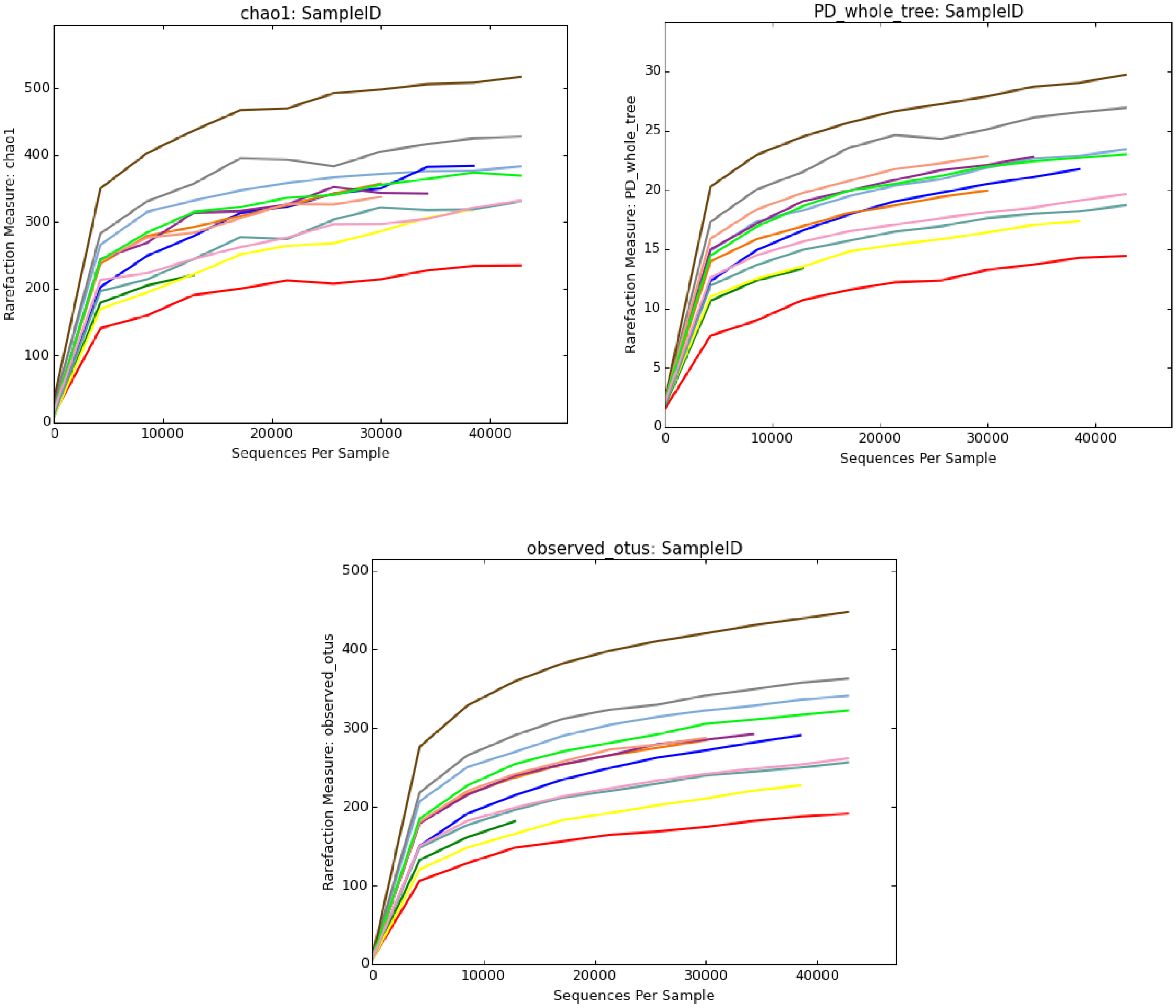

Fig S1: Rarefaction curves.

S2A

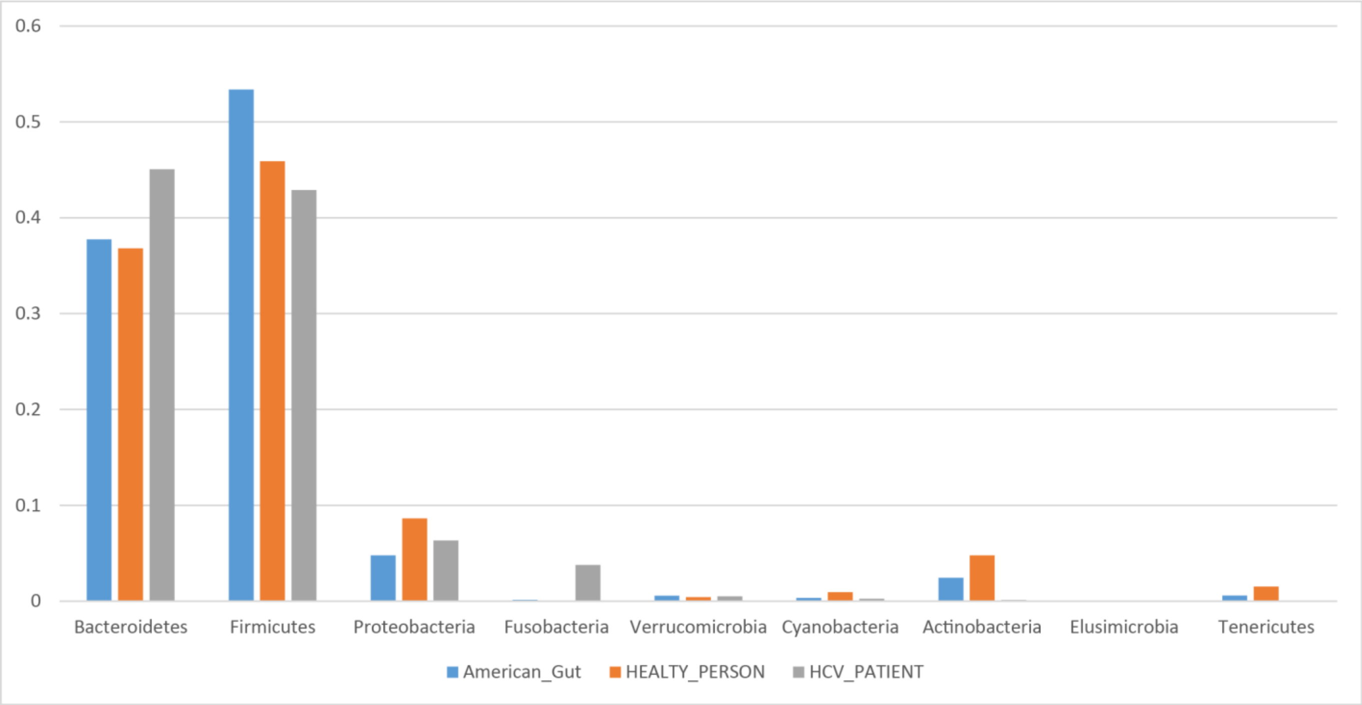

S2B

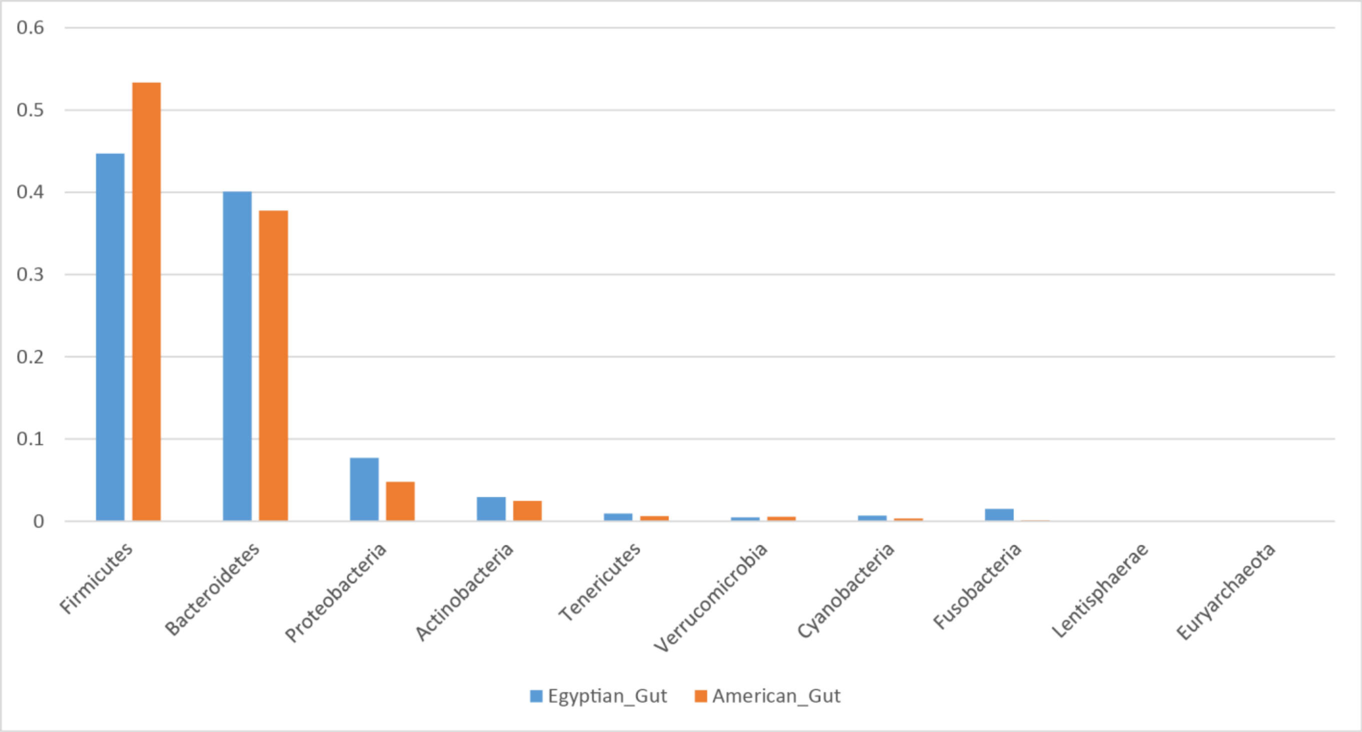

Fig S2A and S2B: Phylum level (American vs. Egyptian Gut microbiome).

S3A

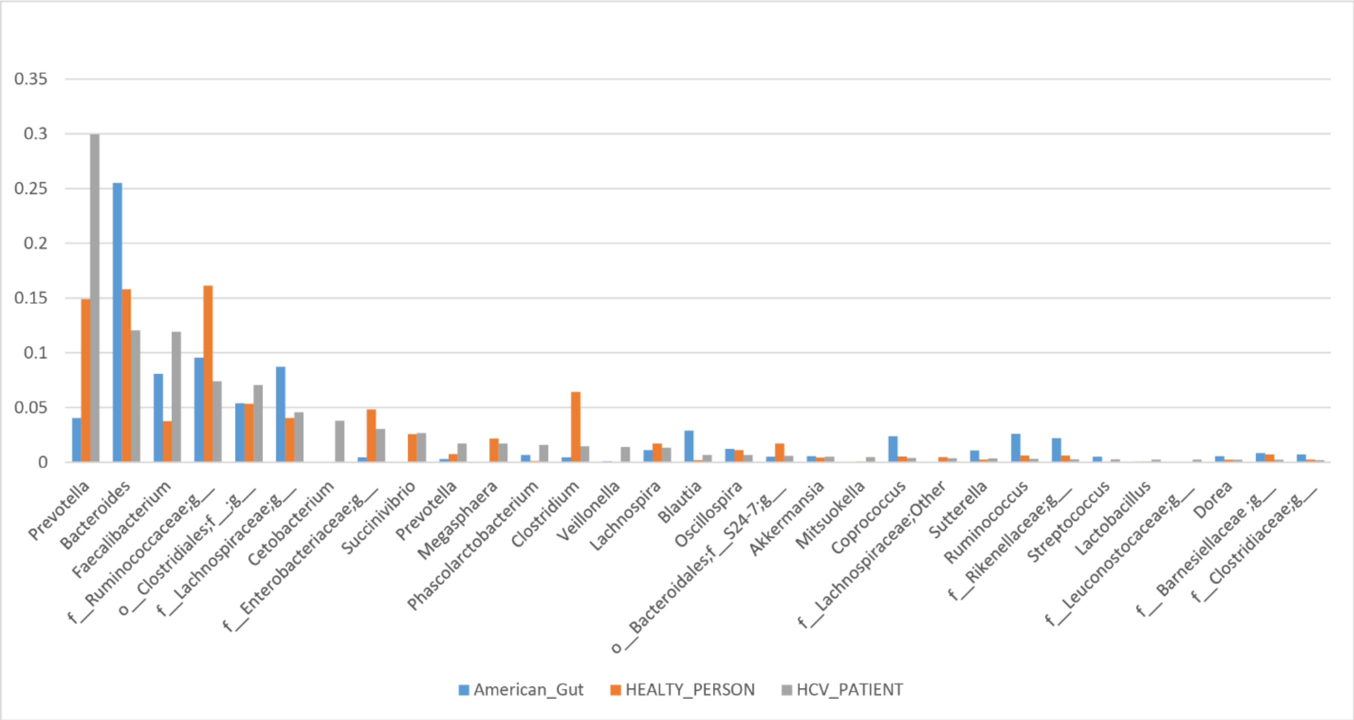

S3B

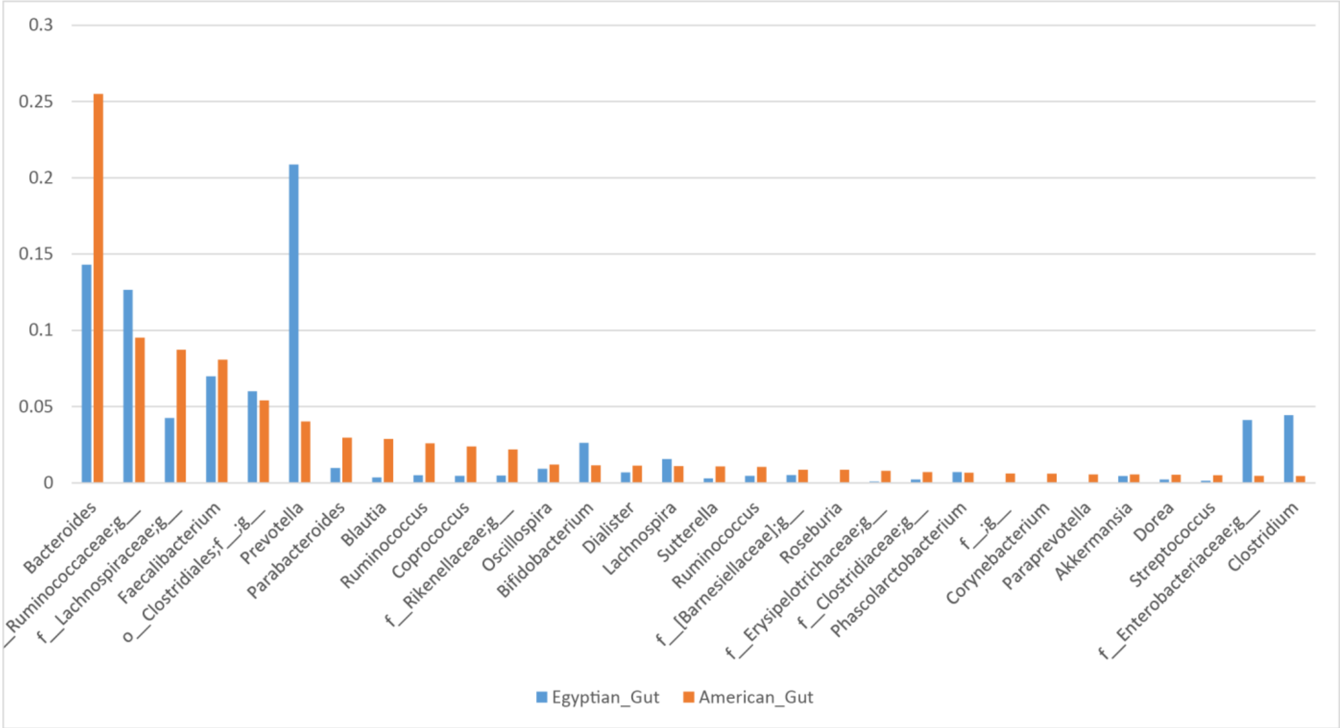

Fig S3A and S3B: Genus level (American vs. Egyptian Gut microbiome). (O_Order, F_Family, G_Genus).

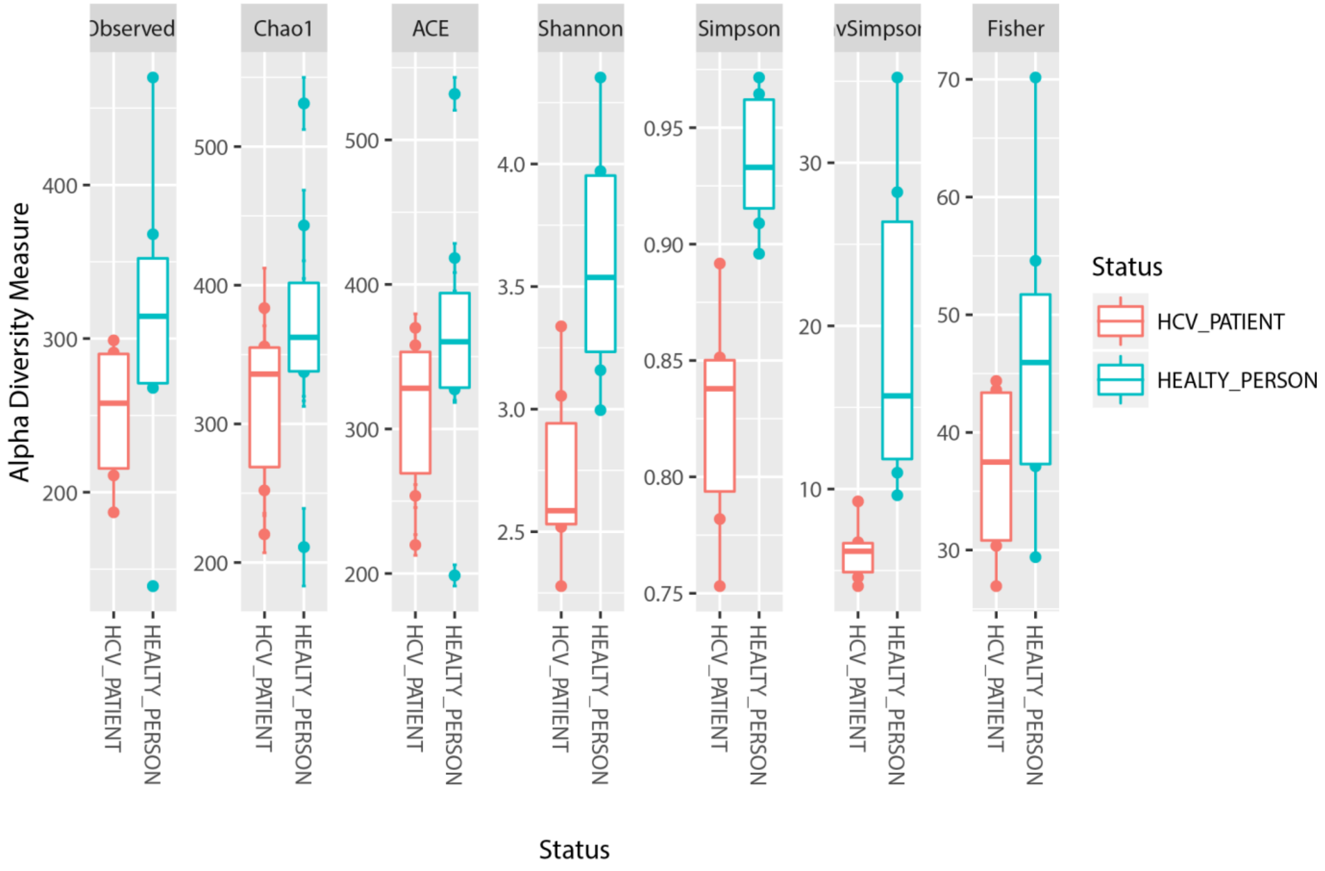

Fig S4: alpha diversity measure using Observed, Chao1, ACE, Shannon, Simpson, vSimpson and Fisher indexes.

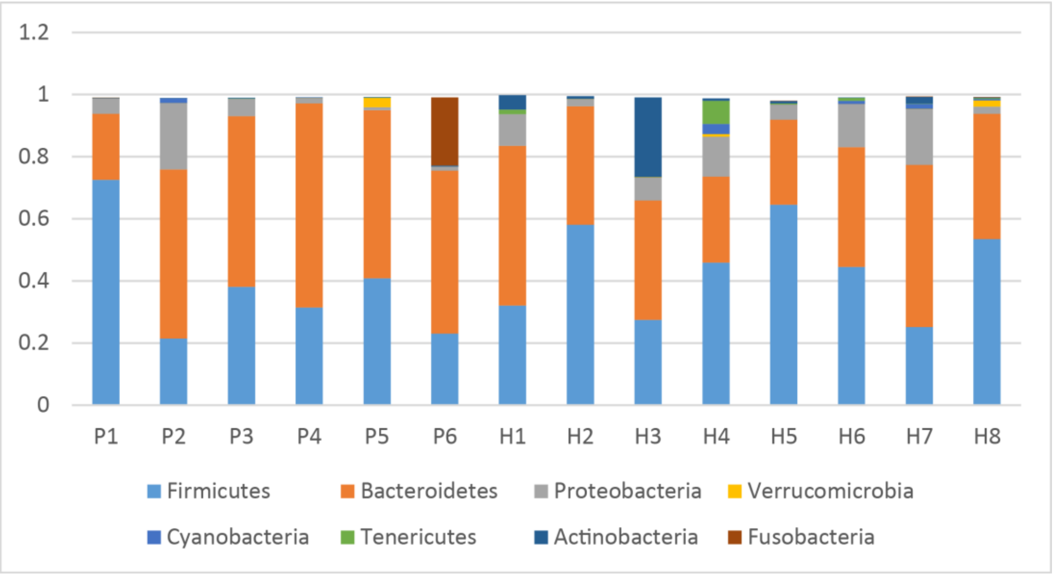

Fig S5: Phylum level representation at all of the samples.

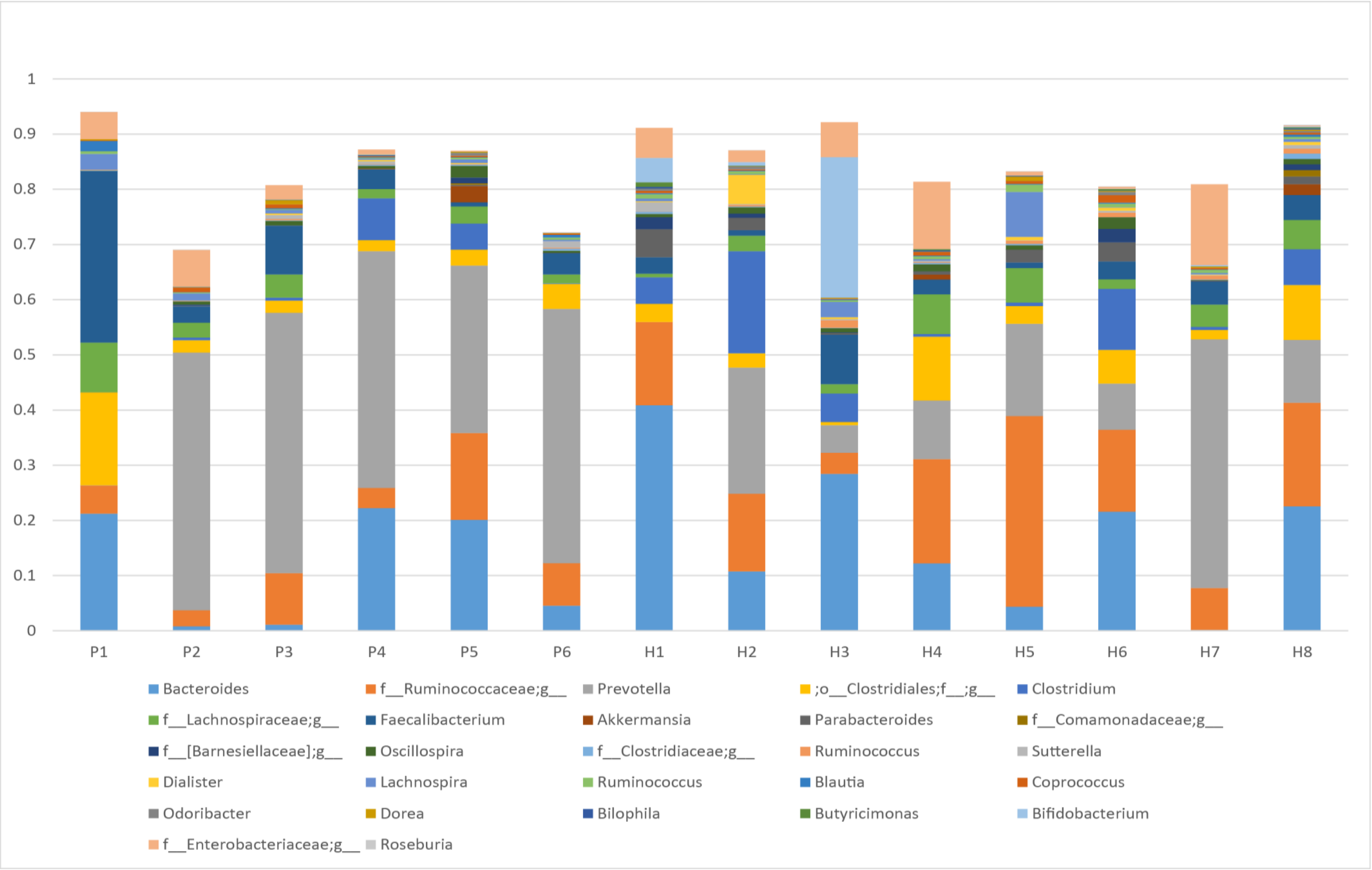

Fig S6: Genus level representation at all of the samples (O_Order, F_Family, G_Genus).
